# Six Year Molars

**Published:** 1873-02

**Authors:** 


### "Six Year Molars."

It is refreshing in these times, when dental literature is not overburdened with good things, to notice, as we did in the January REGISTER, an article with the above caption, leaving proofs of signal triumphs in the preservation of the six year molars. Hitherto, many experienced practitioners of our profession, entertained serious doubts with regard to the practical utility of preserving these organs for any great length of time. But it has been reserved for later skill and more refulgent light from higher realms of dental knowledge, to guide us from the pathway of ignorance and error into one of truth and final triumph.

L. W. Munhall has thrown his spectroscopic beams upon this vexed problem, as would appear from the article to which we refer, and the result of his analysis it will be our purpose to examine.

Articles written in a logical form are universally attractive. Fact or theory supported by all the artfulness of logic are necessarily strengthened, and the inductions which follow, easy and natural. It is a great pity that contributors to dental periodicals, other than L. W. Munhall, do not adopt this convincing method in their compositions. But we tarry too long over these seeming trifles. Let us at once repair to the feast which L. W. Munhall has bidden us: "Two causes," he says, "explain the absence of six year molars from the mouth: first, ignorance and indifference of the parents; second, ignorance and indifference on the part of dentists." Clearly these are two similar propositions so far as parents and dentists are concerned, and both seem charged with the same moral turpitude of offense! Ignorance as here used is not very specific, but employed in a very general sense indeed. Parents may be quite intelligent, yet lacking in information with respect to the preservation of their own and their children's teeth, and still would not fall into the catagory of being ignorant. On the other hand, a dentist whose holy mission is to save every wreck of tooth he sees in children's mouths may be the grossest ignoramus of the dental fraternity. "Out of the mouths of babes shall they be condemned." And the application of these inspired words can be made to dentists who attempt to save six year molars where caries has penetrated to the pulp cavity, with walls thin and badly broken away, with foreign accumulations adherring to the contiguous parts, and the age such as will not warrant protracted operations. Failures will outnumber successes even in the hands of good operators under such circumstances, is a view many take, and it seems well authorized. Of course, there are modifying circumstances, when the dentist should use his best efforts to save these teeth, but these will mainly depend on the age the patient has attained for dental operations, the general characteristics of the teeth, shape and formation of the dental arch, together with the occlusion of the teeth, whether the necessary labor should be expended in attempting to restore these molars. A neophyte in the dental practice can not put his metal to any better test than by applying himself diligently to a few of these cases, and record years afterward his victories.

Ignorance is too general a term to be applicable in the sense in which teeth are lost—at least, this seems to be so with the accumulations of knowledge in this direction, which is at the disposal of every practitioner. Now, to have every body educated and enlightened, including in each one's curriculum, anatomy, physiology, pathology, chemistry, and diettetics generally, so that every mother's son might secure good dentures, would be a most charming society. Unfortunately ignorance prevails. Nor will the time soon come when parents will know anything comparatively about six year molars, or knowing them will take the necessary care for preserving them. The avocations of people are too multiplied and their duties too exacting, to furnish much opportunity for acquiring information respecting the teeth. Usually—there are exceptions—people have sense enough to employ the services of some dentist in whom they have confidence, and then follow his counsels. Nor is it held of high import that every dentist should turn missionary to the cause of his profession, and expound to each of his patients the sublime mysteries of dentistry, however worthy and tempting the field might be. Something rather too much of this is already abroad in the land. Information, pointed and pertinent, communicated quietly to such patients as may desire it, would be well enough, and should be cheerfully given. Beyond this, where some dentists include the whole cyclopedia of dental principles and practice, it is a waste of energy that might be better directed, and not bring reproach upon the profession. Medical practitioners of culture resort to none of these educational plans for the advancement of their patients, nor should dentists.

L. W. Munhall puts in substance a query of this sort: If the six year molars when decayed are extracted merely to give room in the future for other teeth, such a procedure results from ignorance or indifference on the part of the dentist. The fallacy of such a statement will be apparent when an experienced practitioner recalls numerous instances where good results have been attained by removing these teeth. The contracted circles of the maxillaries not admitting the regularity of these teeth, and the difficulties attending the expansion of them by mechanical means, assure us that some cases require this method of extraction as the best remedy that can be employed—"ignorance or indifference" to the contrary notwithstanding.

But we now come to a very interesting case—perhaps he may be some nice little Sunday-school boy, cited by our contributor. Here is his profile: "Herbert E——, aged 12, splendid intellectual head, high frontal and temporal bones, superior maxilliary good, inferior disproportionately small, with chin *retiring*." This good little boy had a bad and ugly tooth, which he took to some dentist, whose cool judgment condemned. Into the tender mercies of L. W. Munhall he then came; "the diseased bone was removed, carbolic acid and iodine were placed into the devitalized pulp cavity, and the tooth filled with gold." A year transpires and the case is seen again, when L. W. Munhall pronounces "the teeth *doing as well as I could wish.*" Most wonderful! Such treatment beyond the beaten tracks of the old masters of the profession is a pleasure to record. Some people who operate on the teeth can be satisfied if the teeth they fill stay in a year, hence they pass judgment on their operations with confidence. There is plenty of time yet for Herbert to come to grief with a swelled molar, and the cervical walls of that filling to bear indications of leakage. Only wait. The difficulties of treating the pulp cavities of the lower molars with success would form an essay of surpassing interest even for the most skilled in our profession. The procedure is attended with something else than carbolic acid and iodine, remedies that have nearly become obsolete. Instead of grappling with each particular step of this most vital part of his treatment, L. W. Munhall has omitted the details, whereby the profession generally could have learned something. This is exceedingly to be regretted. Lastly, we come to the most gushing part of our contributor's article. He says, "he shouldn't hesitate to try and save teeth when they were destitute of bone-making material, decaying rapidly, were frail, and the patient himself hadn't enough phosphorus in his system to keep him in toe nails!" "Bully for him!" With such pluck as that in a fellow, he could buck his head against a stone wall, and never a brain would be knocked out. This sort of vim is good, and not likely to starve. We commend it.

